# ‘Test and Treat’ Among Women at High Risk for HIV-infection in Kampala, Uganda: Antiretroviral Therapy Initiation and Associated Factors

**DOI:** 10.1007/s10461-017-1973-5

**Published:** 2017-11-10

**Authors:** Yunia Mayanja, Onesmus Kamacooko, Daniel Bagiire, Gertrude Namale, Pontiano Kaleebu, Janet Seeley

**Affiliations:** 1 MRC/UVRI Uganda Research Unit on AIDS, Plot 51-59 Nakiwogo Road, Entebbe, Uganda; 2 London School of Hygiene and Tropical Medicine, London, UK

**Keywords:** HIV, High risk women, Test and treat, Anti-retroviral therapy, Uganda (sub Saharan Africa)

## Abstract

Data on implementation of ‘Test and Treat’ among key populations in sub-Saharan Africa are still limited. We examined factors associated with prompt antiretroviral therapy/ART (within 1 month of HIV-positive diagnosis or 1 week if pregnant) among 343 women at high risk for HIV infection in Kampala-Uganda, of whom 28% initiated prompt ART. Most (95%) reported paid sex within 3 months prior to enrolment. Multivariable logistic regression was used to determine baseline characteristics associated with prompt ART. Sex work as main job, younger age and being widowed/separated were associated with lower odds of prompt ART; being enrolled after 12 months of implementing the intervention was associated with higher odds of prompt ART. Younger women, widowed/separated and those reporting sex work as their main job need targeted interventions to start ART promptly after testing. Staff supervision and mentoring may need strengthening during the first year of implementing ‘test and treat’ interventions.

## Introduction

Throughout the world, key populations who include: sex workers, injecting drug users (IDU), men who have sex with men (MSM), transgender people and fisher-folk are disproportionately burdened by the HIV epidemic [1–5]. These key populations and their sexual partners account for 10–51% of new infections in sub-Saharan Africa (SSA) [6–8]. Systematic reviews show that in low and middle income countries (LMICs), female sex workers (FSWs) are 13.5 times more likely to have HIV compared to women in the general population [1]. In Uganda, while the National HIV prevalence has stabilized at 7.3% [9], the prevalence is over 30% among FSWs [10].

The HPTN052 trial showed that immediate ART given to HIV-positive people dramatically reduced HIV-transmission to their HIV-negative partners [11]. These results were received with considerable enthusiasm and the ‘end of AIDS’ heralded [12, 13]. In December 2013, the Ministry of Health (MoH), Government of the Republic of Uganda, issued an ‘Addendum to the National Antiretroviral Treatment Guidelines’ which allowed provision of ART irrespective of CD4 T cell counts to the following HIV-positive groups: pregnant women and breast-feeding mothers, partners in sero-discordant couples, and people with active TB disease or Hepatitis B co-infection with severe chronic liver disease. In addition, it included the recommendation that key populations should initiate prompt ART [14] with the rationale that these groups were making a substantial contribution to new HIV infections. Following the START trial results [15], and the release of the WHO ‘Guidelines on when to start antiretroviral therapy and on pre-exposure prophylaxis for HIV’ [16], the global HIV response is now aimed at reducing HIV transmission by giving prompt ART to all HIV-positive people, a challenging and ambitious goal [17]. This approach is called `Test and Treat’ or `Test and Start’ [18].

The studies that have assessed ‘test and treat’ in sub-Saharan Africa (SSA) have shown that at least half of participants initiate prompt ART [19, 20]. In the PopART trial, the largest so far to deliver ‘test and treat’ at population level, Hayes et al. report less than 45% ART uptake within 6 months with uptake being lower among the younger population [21]. Among MSM in Nigeria prompt ART was associated with disclosure of MSM status [20] while in a cluster randomised trial among the general population in South Africa, participants in the ‘test and treat’ arm were less likely to initiate ART if they had high CD4 T-cell counts, most likely because they were not sick [19]. Studies done among the general population elsewhere, and key populations such as MSM and FSWs also show that being in a healthy state [22–25] is associated with lower ART uptake. Fear of side effects [25, 26] has been reported as another barrier to prompt ART.

The enthusiasm for `Test and Treat’ has been tempered by a growing recognition of the challenges that impede progress in providing treatment to everyone living with HIV [21, 27–31] and achieving the second ‘90’ of the UNAIDS 90–90–90 targets. Despite these challenges, concerns over the spread of HIV [32] and the prospect of increased ART drug resistance in Africa [33] have increased efforts to facilitate the roll-out of treatment and identify barriers to initiating prompt ART. We build on work done in the general population [19, 21] and MSM [20] to determine factors associated with prompt ART initiation among FSWs, who are criminalized in many parts of SSA and in whom the dynamics of treatment uptake may differ from the general population and settings that have decriminalized sex work. We hypothesize that treatment uptake will be different from that seen in the general population. We use data collected over 2 years and 8 months in a ‘Test and Treat’ intervention in Kampala, Uganda.

## Methods

### Study Design and Setting

We performed a retrospective cohort analysis of participant records within a research clinic implementing ‘Test and Treat’ among women at high risk for HIV infection, who attended the clinic between August 2014 and April 2017. The clinic (Good Health for Women Project Clinic-GHWP) was established in a peri-urban community in southern Kampala in 2008. Women attending the clinic engage in sex with men for money, goods or favors; the recruitment of women from commercial hotspots has been described elsewhere [10].

Women are enrolled into the GHWP clinic irrespective of HIV status, and invited for quarterly visits for HIV prevention and care services. At enrolment, women are offered HIV counselling and testing (HCT) and receive same day results. HIV-positive participants received free HIV care including Tuberculosis screening and treatment, prophylaxis for opportunistic infections (OIs) and ART. ART was initiated using CD4 + T-cell counts (< 350 cells/µl) and WHO clinical stage criteria until August 2014 when the clinic implemented ‘Test and Treat’ in line with the new MoH guidelines.

Repeat HCT was done at quarterly follow up visits only for women who previously tested HIV-negative. The women received free services including treatment for common illnesses, contraception, syndromic management of sexually transmitted infections (STIs), counselling for alcohol use disorders, male and female condoms and treatment for their children below 5 years. They were encouraged to refer their male regular partners to the clinic for HIV prevention, care and treatment interventions. Enrolment of women continued throughout the study period and field workers maintained contact details of enrolled participants.

### Study Participants, Eligibility Criteria

We selected participants from all HIV-positive ART-naïve women who were newly enrolled in the clinic from August 2014 to April 2017 and who were ≥ 18 years. In addition, all HIV-negative women who changed to HIV positive status (HIV seroconversion) during the study period became eligible.

### ART Preparation, Initiation and Follow up Process

Study counsellors gave group information sessions about the ‘Test and Treat’ guidelines to participants and the benefits to their health and the wider community. Women were also counselled on disclosure, drug reactions and adherence, and then individually assessed for readiness to start ART using the ART readiness checklist. Women who were ready, initiated ART on the same day. Those who opted out of same-day initiation continued to be counselled, and a plan made with the aim of initiating ART within 7 days for pregnant women, and within 1 month for the others. Women were considered to have initiated prompt ART if they started treatment within 1 month or 1 week (if pregnant) of an HIV-positive diagnosis as per the “Consolidated guidelines for prevention and treatment of HIV in Uganda” [34]. Counselling sessions during clinic visits continued for those who did not initiate prompt ART. They continued to be followed up at the clinic to receive free services and treatment of other illnesses, and could initiate ART when they were ready. The women initiated first line treatment composed of two nucleoside reverse transcriptase inhibitors (NRTIs) and one non-nucleoside reverse transcriptase inhibitor (NNRTI); they were initially given 2 week’s treatment and scheduled for follow up 2 weeks later but were free to return earlier if they experienced side effects or other challenges. Women were then given treatment for 1 month, and the interval increased to 2 months’ treatment if they had no clinical events or adherence problems. Those who adhered and tolerated the drugs well were then given 3 months of treatment and scheduled for quarterly visits onwards. ART was provided free of charge throughout the study period. The women gave blood samples for laboratory monitoring according to the MoH guidelines. CD4 + T-cell counts were tested at baseline and every 6 months, and viral load monitoring after at least 6 months on ART.

### Study Outcomes

*Prompt ART initiation* was defined as dispensing initial ART to an eligible participant within 1 month of an HIV-positive diagnosis or within 1 week if pregnant.

Independent variables were collected at the time of HIV diagnosis and included: socio-demographic variables (age, marital status, education level, religion, main job); behavioral variables included: paid sex, substance use, contraceptive use and sexual partner violence in the 1 months prior to enrolment; condom use 1 month prior to enrolment and history of ever testing for HIV); one biological variable (CD4 T-cell count). We also included a time variable that measured the duration between ‘test and treat implementation and becoming eligible.

### Data Collection

Data on socio-demographics, sexual behavior, prior HIV testing, substance use, contraception and sexual partner violence were collected using electronic (MS Access) interviewer administered questionnaires during one on one private sessions held in clinic rooms with trained study counsellors. Alcohol use was assessed by using the Alcohol Use Disorders Identification Test (AUDIT) questionnaire which consists of 10 questions to assess alcohol use disorders. Alcohol use was classified into two categories which included: low risk drinkers: score 0–7 and high risk drinkers: score 8 + . Data on ART start dates, duration on ART and prescriptions were recorded by clinicians. HIV results were recorded on laboratory source documents by laboratory staff while CD4 results were generated as print outs. Data on HIV results and ART were entered into OpenMRS by data management assistants.

### Laboratory Methods

HIV testing was performed on serum using two or more rapid diagnostic tests for antibodies to HIV. Screening was performed using Determine. A non-reactive test result was given to the participant as HIV-negative while a reactive test was confirmed using Statpak. A reactive Statpak result was given to the participant as HIV-positive. Discordant determine and Statpak results, were subjected to a tie breaker test using Unigold. The result given by Unigold was taken as final for presence or absence of antibodies to HIV and results given to the participant.

CD4 testing was performed on plasma using Multi-test Trucount tube CD4/CD8/CD3-Lyse no wash (BD. USA).

### Statistical Analysis

The MS Access and OpenMRS databases were merged into one database, cleaned, and exported to STATA 12.1 (StataCorp, College Station, TX, USA) for analysis. Prompt ART initiation was a binary outcome (*Yes* initiated prompt ART; *No* did not initiate prompt ART). Participants’ categorical demographic and behavioral characteristics were summarized by counts and percentages. Continuous characteristics were summarized by means and standard deviations (SD) or medians and inter quartile ranges. We determined the proportion who initiated prompt ART as the number that started ART within 1 month of eligibility (non-pregnant) or within 1 week (pregnant) divided by the total number eligible, and expressed as a percentage. The proportion who initiated prompt ART was further analyzed by the different demographic and behavioral characteristics using Chi square tests. Logistic regression models were fitted to find factors associated with prompt ART initiation at unadjusted analysis; those for which the association attained statistical significance on log likelihood ratio test (LRT), p < 0.20 were selected for the multivariable logistic regression model. We used complete case analysis to run our final model. Age was considered as a priori confounder. Factors were retained in the final multivariable logistic regression model if their inclusion did not make the model significantly worse at p < 0.05. Results are presented as adjusted odds ratios (AOR) with p values and 95% confidence intervals (CI).

## Results

### Recruitment Profile

**Fig. 1:**
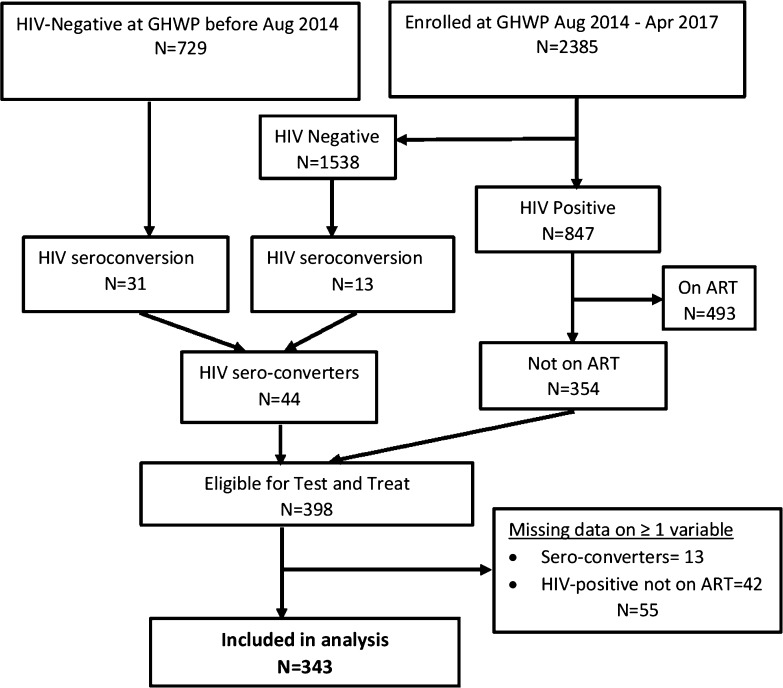
Screening profile of study participants

The recruitment profile for participants is summarized in Fig. 1. From August 2014 to April 2017, we enrolled 2385 women at high risk of HIV-infection at the GHWP clinic. Of these 847 were HIV-positive of whom 354 were not yet on ART and were therefore eligible for ‘test and treat’. In addition, women joined the `test and treat cohort’ during the study period, because 44 participants who were HIV-negative became HIV-positive during the study period. Therefore, a total of 398 (354 + 44) participants were eligible, of whom 343 had complete data for all variables and were included in our final analysis.

### Characteristics of Study Participants

The mean (± SD) age of study participants was 28.9 ± 6.1 years. The majority (73.5%) were widowed or separated, 60.4% had primary level education and 65.6% were Christian. The median (IQR) CD4 + T-cell count at enrolment was 544 (327–775) cells/µl. Ninety-five percent of participants reported engaging in paid sex in the 3 months prior to enrollment. Overall, 65.3% of participants reported sex work as their main job, 20.1% mainly worked in entertainment facilities (bars, night clubs and karaoke) and the rest reported having other main job(s) such as the hospitality industry (restaurants, beauty and massage parlors, guest houses) or no job. High risk alcohol consumption was reported by 61.5% of all participants. Thirty-five percent consumed drugs, the most common being *kuber*/smokeless chewing tobacco and khat. Sexual partner violence was reported by 48.7% and was perpetrated by both casual partners and marital partners. Details are shown in Table 1.

**Table 1:** Sociodemographic and behavioural characteristics of study participants

Characteristic	Categories	Frequency (n = 343)	Percentage (%)
Age	< 25	90	26.2
	25–29	111	32.4
	30–34	71	20.7
	≥ 35	71	20.7
Education level	No education	31	9.0
	Attended primary	207	60.4
	Attended Secondary or higher	105	30.6
Current marital status	Never married	67	19.5
	Married	24	7.0
	Widowed/Separated	252	73.5
Religion	Christian	225	65.6
	Muslim and other	118	34.4
Main job	Sex work	224	65.3
	Entertainment	69	20.1
	No job/other job	50	14.6
Paid sex in past 3 months	Yes	324	94.5
	No	19	5.5
Drinking pattern (AUDIT score)	Low risk drinkers	132	38.5
	High risk drinkers	211	61.5
Sexual partner violence in past 3 months	Yes	167	48.7
	No	176	51.3
HIV sero-conversion	Yes	31	9.0
	No	312	91.0
Ever tested for HIV	Yes	298	86.9
	No	45	13.1
Drug use in past 3 months	Yes	120	35.0
	No	223	65.0
Time since T&T implementation	12 months	112	32.7
	> 12 months	231	67.3

### Factors Associated with Prompt ART Initiation

A total of 96 (28%) participants initiated prompt ART during the study period, the median time to ART initiation being 6 days (IQR 0–14 days). At bivariate analysis, prompt ART initiation was less likely among women who reported sex work as their main job (OR sex work_vs_other job or no job: = 0.48, 95% CI 0.30–0.79), and young women (OR < 25_vs_ ≥ 35: = 0.39, 95% CI 0.20–0.77) and (OR 25–29_vs_ ≥ 35: = 0.44, 95% CI 0.23–0.83). Prompt ART initiation was more likely among those who became eligible after 12 months of implementing the intervention (OR 1.79, 95% CI 1.05–3.06) compared to those who became eligible during the first 12 months.

At adjusted analysis, when compared with women aged ≥ 35 years, prompt ART initiation was less likely among young women aged < 25 years; (AOR0.28, 95% CI 0.13–0.61) and those aged 25–29 years (AOR 0.40, 95% CI 0.20–0.79). Prompt ART was also less likely among women who reported sex work as their main job (AOR 0.44; 95% CI 0.25–0.78) compared to those reporting other job(s) or no job. Regarding marital status widowed/separated women were less likely to initiate prompt ART (AOR 0.45; 95% CI 0.23–0.86) compared to never married women. Those who became eligible for the ‘test and treat’ intervention after 12 months of its implementation were more likely to initiate prompt ART (AOR 2.58; 95% CI 1.41–4.71) compared to those who became eligible during the first 12 months of the intervention as shown in Table 2.

**Table 2:** Multivariable model for factors associated with prompt ART initiation

Characteristic	N (col %)	Prompt ART N (row %)	Unadjusted OR (95% CI)	LRT P value	Adjusted OR (95% CI)	P value
	**343**	**96(28)**				
Age				0.03		
≥ 35	71(21.0)	30(42.3)	1		1	
< 25	90(26.0)	20(22.2)	0.39(0.20–0.77)		0.28(0.13–0.61)	0.001
25–29	111(32.0)	27(24.3)	0.44(0.23–0.83)		0.40(0.20–0.79)	0.009
30–34	71(21.0)	19(26.8)	0.50(0.25–1.01)		0.54(0.26–1.16)	0.116
Current marital status				0.106		
Never married	67(19.5)	23(34.3)	1		1	
Married	24(7.0)	10(41.7)	1.37(0.53–3.55)		0.80(0.28–2.34)	0.69
Widowed/separated	252(73.5)	63(25.0)	0.64(0.36–1.14)		0.45(0.23–0.86)	0.016
Religion				0.441		
Muslim and other	118(34.4)	30(25.4)	1			
Christian	225(65.6)	66(29.3)	1.22(0.74–2.02)			
Education level				0.935		
No education	31(9.0)	9(29)	1			
Primary	207(60.4)	59(28.5)	0.97(0.42–2.24)			
Secondary or higher	105(30.6)	28(26.7)	0.89(0.37–2.16)			
Main job				0.004		
No job/other jobs	119(34.7)	45(37.8)	1		1	
Sex work	224(65.3)	51(22.8)	0.48(0.30–0.79)		0.44(0.25–0.78)	0.005
Paid sex in past 3 months				0.174		
No	19(5.5)	8(42.1)	1		1	
Yes	324(94.5)	88(27.2)	0.51(0.20–1.32)		0.89(0.30–2.69)	0.838
Drinking pattern (AUDIT score)				0.083		
Low risk drinkers	132(38.5)	44(33.3)	1		1	
High risk drinkers	211(61.5)	52(24.6)	0.65(0.41–1.06)		0.61(0.35–1.04)	0.072
Drug use in past 3 months				0.722		
Yes	120(35.0)	61(27.3)	1		1	
No	223(65.0)	35(29.2)	1.09(0.67–1.79)		1.50(0.85–2.66)	0.161
Sexual partner violence in past 3 months				0.167		
No	176(51.3)	55(31.2)	1		1	
Yes	167(48.7)	41(24.6)	0.72(0.45–1.15)		0.67(0.39–1.14)	0.137
HIV sero-conversion				0.775		
No	312(91.0)	88(28.2)	1			
Yes	31(9.0)	8(25.8)	0.89(0.38–2.05)			
Ever tested for HIV				0.235		
No	45(13.1)	16(35.6)	1			
Yes	298(86.9)	80(26.9)	0.67(0.34–1.29)			
Time since T&T implementation				0.029		
≤ 12 months	112(32.7)	23(20.5)	1		1	
> 12 months	231(67.3)	73(31.6)	1.79(1.05–3.06)		2.58(1.41–4.71)	0.002

## Discussion

We found that a low proportion of women initiated prompt ART compared to other studies that have examined the delivery of the test and treat intervention [19–21]. Iwuji and colleagues, for example, report high ART uptake, however this was only observed in those who linked to care [35]; overall, ART uptake in that trial was still low considering that almost half of HIV-positive individuals did not link to care within 6 months. We report prompt ART as initiating within 1 month while others [20, 21, 35] have reported initiation between 3 and 6 months. The shorter duration may further explain lower uptake in our study.

Women who reported sex work as their main job were less likely to initiate prompt ART compared to those who reported other jobs or no job. Structural and social barriers may hinder prompt ART initiation among FSWs in our setting where sex work is still criminalized. Communities worldwide still exhibit varied levels of stigma and discrimination against FSWs [36, 37]. To avoid these negative consequences, many FSWs do not reveal their HIV-positive status to community members. Many do not want to be seen with pills because taking ART is one way of exposing their HIV-positive status making them lose clients and money [38]. The lifestyle of FSWs also poses barriers to prompt ART initiation because of working hours which do not allow time to make initial clinic visits to start and stabilize on treatment, and fear of being isolated from the FSW support structures if found taking ART [38, 39]. Increased efforts are needed to create a sex worker-supportive environment and partnerships using community mobilization among FSWs in order to facilitate universal access to HIV prevention services and identify acceptable ART delivery strategies [40].

In our study women aged less than 30 years had a lower likelihood of prompt ART initiation; the odds were however lower among young women (< 25 years) compared to those aged 25–29 years. Studies done among key populations elsewhere [24, 41–43], and the general population have also found prompt ART initiation less likely among young people. Initiation of prompt ART at a young age means, potentially, many years on ART; this requires a lifelong commitment which may not be a priority for young HIV-positive people who are not sick. In addition, young people are not fully economically independent and may not have resources to support clinic visits for ART initiation and early follow required before they are stable on treatment. In sex work settings, younger HIV-positive women experience more unequal power relations, gender inequities, limited social support and intimate partner violence when compared to older women [44], which affects their ability to engage with community social services including initiating prompt ART [45].

We observed that ART initiation was less likely among widowed and separated women compared to those who were never married. Similar findings have been reported from the general population in sub-Saharan Africa [46, 47] and elsewhere [48]. Widowhood and divorce/separation are still associated with stigma and shame in many developing communities [49, 50]. Widows and separated/divorced women may experience rejection and violence from in-laws and former spouses [49–52]. This social marginalization coupled with engagement in sex work may further reduce their opportunities for a social support system that facilitates disclosure of HIV status and the subsequent decision to initiate ART.

We found that women who were more recently enrolled in the cohort (after 12 months of implementing ‘test and treat’), were more likely to initiate prompt ART. This finding is consistent with other studies [22, 53, 54]. We attribute the higher likelihood of prompt ART initiation in later months to increased staff confidence and experience with the new ART initiation guidelines. It is also likely that the intervention was more popular among the women after 1 year of implementation increasing the likelihood of prompt ART initiation. We however note, that even though prompt ART initiation was more likely among the recently enrolled women, the proportion who initiated was still very low suggesting that staff experience alone does not lead to improved ART uptake.

### Study Limitations

One of our main limitations was missing data on one or more variables for 55 women that were excluded from the final analysis. This could have reduced our power of predicting prompt ART initiation. We did not assess for health facility factors, the study staff received training before and during implementation of test and treat, drugs, clinic and laboratory supplies and registers for documentation were always available. Women received comprehensive health care in addition to HIV care and treatment; the role of the health facility could have been limited. Furthermore, we did not collect data on the reasons for non-uptake of prompt ART for those that did not initiate. Such data could inform future strategies aimed at improving prompt ART among key populations.

## Conclusion

We found a low uptake of prompt ART among FSWs. Our findings provide information that highlights the need to target young women, those whose main job is sex work, and the widowed/separated for prompt ART initiation. FSWs should be involved in the design and implementation of tailored interventions that provide an enabling environment to support them. This approach together with community engagement activities through peer educators and health workers enables close follow up of all HIV positive FSWs for purposes of continued education about prompt ART and could play an important role in prompt ART initiation and increased ART coverage in this population. In addition, regular supervision and mentoring activities for clinical care need more support during the first year; they include but are not limited to: staff training, ART knowledge and skills assessment, document review to ensure accurate and consistent data capture and participant satisfaction surveys.
